# Correlations between muscle strength and psychological health in Chinese adolescents: a cross-sectional analysis

**DOI:** 10.7717/peerj.14133

**Published:** 2022-10-06

**Authors:** Jinkui Lu, Hao Sun, Ningling Liu, Jianhua Qiu, Xiaofei Xia

**Affiliations:** 1 School of Physical Education, Shangrao Normal University, Shangrao, China; 2 College of Physical Education, Jiangxi Normal University, Nanchang, China

**Keywords:** Muscle strength, Psychological symptoms, Correlational analysis, Mental health, Chinese adolescents

## Abstract

**Background:**

Studies indicate that muscle strength is associated with good mental health. However, it remains unclear whether muscle strength is directly correlated with psychological symptoms in Chinese adolescents. Given the declining muscle strength and worrying mental health status of Chinese adolescents, the present study aimed to estimate the correlation between muscle strength and psychological symptoms as well as explore the gender differences in those correlations in Chinese adolescents.

**Method:**

From April to July 2018, a total of 14,344 Chinese adolescents from eight provinces were selected using a stratified clustered sampling method. Psychological symptoms were evaluated using the Multidimensional Sub-health Questionnaire of Adolescents (MSQA), a verified and validated questionnaire that assesses three psychological areas: emotional symptoms, behavioral symptoms, and social adaptation difficulties. Muscle strength was assessed using grip strength, sit-ups, and standing long jump. The Chi-square test was used to compare the detection rates of the different categories of psychological symptoms. A logistic regression analysis was used to analyze the correlations between muscle strength and psychological symptoms and explore the gender differences in those correlations in Chinese adolescents.

**Results:**

In general, the detection rate of psychological symptoms for Chinese adolescents was 21.39%. Males had a higher detection rate of psychological symptoms (22.12%) than females (20.66%, *p* < 0.05). Psychological symptoms were present in significantly fewer Chinese adolescents with a muscle strength index >P75 (19.26%) than among Chinese adolescents with a muscle strength index ≤P25 (23.00%) (χ^2^ = 23.417, *p* < 0.01). Compared with females, the OR values for males in most groups were over one (OR = 1.04–1.43), indicating that males have a higher risk of psychological symptoms than females.

**Conclusions:**

The psychological symptom detection rate of Chinese adolescents is correlated with muscle strength. Psychological symptoms were more correlated to muscle strength in males than in females. The significance of the present study lies in the important insights for integrated mental and physical fitness intervention strategies that promote muscle strength and psychological symptoms simultaneously.

## Introduction

Adolescence is a difficult stage of life involving profound individual, biological, and social changes (Sawyer et al., 2012; Viner et al., 2012; Gore et al., 2011; Patton & Viner, 2007). Muscle strength development during adolescence is an important component of physical fitness and is a predictor of health in early adulthood (Kawada, 2020; Garcia-Hermoso, Ramirez-Campillo & Izquierdo, 2019). The 2018 Physical Activity Guidelines for Americans recommend teenagers participate in muscle resistance exercises at least three times a week for a better, healthier life (Piercy et al., 2018). Studies have shown that muscle strength in children and adolescents is a predictor of all-cause mortality, chronic cardiovascular disease, and even psychological health (Zhang et al., 2022; Smith et al., 2014; Perez-Bey et al., 2018; Ludyga et al., 2021; Rodriguez et al., 2015). It was also reported that muscle strength in childhood has been shown to track into adulthood (Castillo-Garzon et al., 2007) and is linked to future cardiovascular disease (CVD) risk (Grontved et al., 2015).

Adolescents are prone to a variety of psychological symptoms such as non-suicidal self-harming behaviors, depression, hostility, and anxiety (Schoeps et al., 2018; Kieling et al., 2011; Perou et al., 2013; Resnick et al., 1997). According to relevant studies, about 10–20% of teenagers have at least one psychological symptom (Younger, 2016; WHO, 2020). More than 50% of adults have been diagnosed with some kind of mental illness, and the majority of symptoms reported in adulthood can be traced back to before the age of 14 years old (Xu et al., 2019).

A study has suggested that muscle strength is associated with mental health outcomes in adults (Hwang & Ahn, 2021). A narrative review summarized that all eight cross-sectional studies included in the review reported significantly lower odds of having depressive symptoms with increased levels of muscular strength. This association persisted even after adjusting for several confounders including the level of physical activity, particularly in older people (Volaklis et al., 2019). A longitudinal cohort study identified the bidirectional associations between handgrip strength and depressive symptoms in Chinese adults (Lian et al., 2021). Low handgrip strength has also been found to be associated with poor mental health among Korean males (Hwang, Ahn & Choi, 2021). However, it remains unclear whether muscle strength is directly linked to psychological symptoms in Chinese adolescents.

Given the declining muscle strength (Dong et al., 2021; Department of Physical Health & Arts Education Ministry of Education, 2021) and mental health status of Chinese adolescents (Xin, Niu & Chi, 2012; Zhang et al., 2020a), the present study aimed to explore the correlation between muscle strength and psychological symptoms and explore the gender differences of those correlations in Chinese adolescents. We hypothesize that muscle strength is negatively correlated with the presence of psychological symptoms in Chinese adolescents and that psychological symptoms in males were more correlated to muscle strength than in females.

## Materials and Methods

### Data source and participants

**Figure 1:**
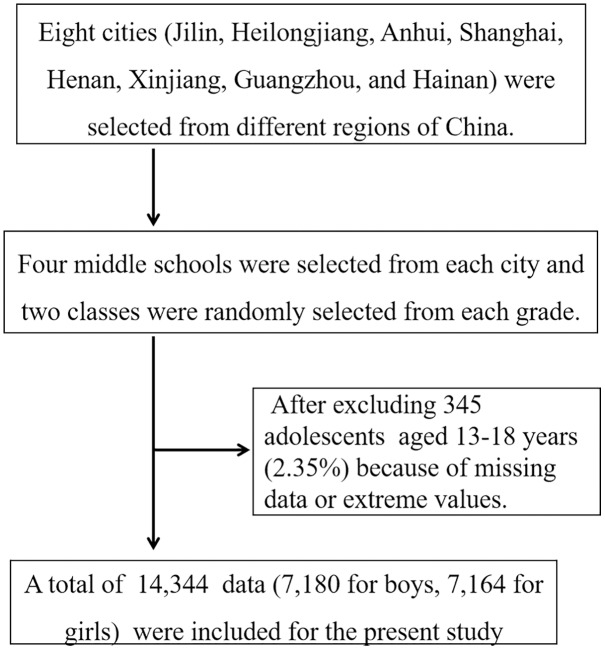
The flow of the participants in the trial.

The study was conducted from April to July 2018. All students included in this study met the following conditions: (1) current student enrolled in school; (2) students of average intelligence as judged by the class teacher, and physically able to participate in the muscle strength test; (3) and voluntarily willing to participate in this study. Eight cities (Jilin, Heilongjiang, Anhui, Shanghai, Henan, Xinjiang, Guangzhou, and Hainan) were selected from different regions of China and four middle schools were selected from each region. Two classes were randomly selected from each grade and the eligible students in the selected class were recruited as participants. In total, 14,689 Chinese adolescents were surveyed by questionnaire and 14,344 valid responses were collected. The valid response rate was 97.65%, with a total of 7,180 male students and 7,164 female students submitting valid responses (Fig. 1). The age range of the surveyed students was between 13–18 years old and the average age was 15.51 ± 1.72 years old. The survey was approved by the Ethics Committee of the School of Physical Education, Shangrao Normal University, and was carried out after approval (2018R-0219). The study was conducted following the requirements of the World Medical Association Declaration of Helsinki. Written consent was obtained from the student participants and the schools and parents of the participating students.

### Demographic information and general household survey

The questionnaire was compiled by experts after a thorough literature review, followed by a discussion and analysis of the results (Tao, 2017). Demographic information obtained from the general household survey included: age, gender, grade level, the region and city (town) of the surveyed participants, the vocations and educational backgrounds of their parents, and annual family income.

### Muscle strength test

The muscle strength test included three items: a grip test to measure upper limb strength, sit-ups to measure torso strength, and a standing long jump to measure lower limb strength. The tests were administered following the methods required by the National Student Physical Health Survey (CNSSCH Association, 2007). The instruments were calibrated before use every day by the PE teachers. These teachers filled in the test results on the test cards of the students. Grip strength was tested by an electronic grip dynamometer (CAMRY EH101, Guangdong, China) to 0.1 kg, the students were required to stand relaxed with arms naturally drooping, and hold the grip dynamometer in one hand with full force for at least 2 s. The test was conducted twice and the larger value was recorded as the result. Standing long jump was tested after preparing for the ankle joint and knee joint. The students stand on the starting line with two-foot shoulders-with apart and jump out from the starting line as best as they can. The distance between the starting line with the heel of the foot closest to the starting line was recorded as 0.1 cm. Each student tried three times and the larger value was recorded as the result. Sit-up was tested on a yoga mat. Students lie on their back with their legs naturally bent, hands by their ears. After receiving the start signal, they sat up with their forehead touching their knees and went back to the start position repeatedly for 60 s. The number of times the hands touched the knees was recorded as the result. The result was standardized to obtain the Z value for the three muscle strength tests. For example, Z_grip strength_ = (actual test score − national average score of the age)/national standard deviation of the age. The average score by age and the national standard deviation by age were obtained from the Chinese National Survey on Students’ Constitution and Health in 2014 (CNSSCH Association, 2007). The muscle strength index was calculated by adding the three test Z scores together: Muscle strength index = Z_grip strength_ + Z_sit-ups_ + Z_standing long jump_. The muscle strength level was then used to divide the study participants into four groups by quartile (≤P25, P26–50, P51–75, >P75) and the presence of psychological symptoms within each group was then compared.

### Survey of psychological symptoms

The Multidimensional Sub-health Questionnaire of Adolescents (MSQA) by Tao et al. (2008) was used to measure the psychological symptoms of Chinese teenagers in this study. This questionnaire has 39 entries grouped into three topic areas: emotional symptoms (eight entries), behavioral symptoms (eight entries), and social adaptation difficulties (13 entries). Each entry has six options to describe the duration of psychological symptoms and the participants choose one option that can best reflect their status. Options that described the duration over one month (*e.g.*, more than 2 months) were recorded as 1 score. The remaining options were recorded as 0 scores. If the scores reached 3, 1, and 4 for the three topic areas, the students were defined as having emotional symptoms, behavioral symptoms, and social adaptation difficulties respectively. The total score was obtained by adding the scores of the three topic areas up. If the total score was ≥8, the students were defined as having psychological symptoms. This scale has been validated by many studies (Zhang et al., 2020b; Tao et al., 2020). The questionnaire has good credibility and the Cronbach α coefficient of the scale is 0.963 (Xing et al., 2008; Qi et al., 2008). The authors also obtained permission to use this instrument from the copyright holders.

### Quality control

Trained specialists were asked to survey the students. A total of six to eight specialists were assigned to each of the four groups and the surveys of the students were simultaneously conducted at schools in different cities. The questionnaires were filled out anonymously. The students were asked to fill them out independently after school and the questionnaires were retrieved on site. Any incorrect or missing fields were completed by the students to ensure the validity and completeness of the questionnaire.

### Stats and analysis

The χ^2^ value was used to compare the detection rates of the psychological symptoms of Chinese adolescents. The presence of psychological symptoms was used as the dependent variable in the logistic regression analysis of the muscle strength of Chinese adolescents and psychological symptoms. The logistic regression analysis was conducted to explore the gender differences between males and females on psychological symptoms influenced by muscle strength. The data were entered twice using EpiData3.1 (EpiData Association, Odense, Denmark) and analyzed with SPSS25.0 (SPSS Inc., Chicago, IL, USA) and *α* = 0.05 was set as the two-sided test level.

## Results

Table 1 shows that the psychological symptom incidence among the Chinese adolescents studied was 21.4%. Among the three psychological areas, the detection rate of emotional symptoms (27.7%) and behavioral symptoms (27.5%) was higher than that of social adaptation difficulties (17.1%). Males (22.1%) had a higher detection rate of psychological symptoms than females (20.7%, χ^2^ = 4.535, *p* = 0.033). The detection rate of psychological symptoms was 19.3% for adolescents with a muscle strength index >P75, which was significantly lower than among those with a muscle strength index ≤P25 (23.0%, χ^2^ = 23.417, *p* < 0.001).

**Table 1:** Comparison of psychological symptoms of Chinese adolescents in different categories.

Category	*N*	Emotional symptoms			Behavioral symptoms			Social adaptation difficulties			Psychological symptoms		
		Prevalence	*χ* ^2^	*p*	Prevalence	*χ* ^2^	*p*	Prevalence	*χ* ^2^	*p*	Prevalence	*χ* ^2^	*p*
Gender													
Boys	7,180	1,996 (27.8)	0.108	0.743	2,038 (28.4)	6.061	0.048	1,308 (18.2)	12.150	0.000	1,588 (22.1)	4.535	0.033
Girls	7,164	1,974 (27.6)			1,902 (26.6)			1,148 (16.0)			1,480 (20.7)		
Grade													
Junior high school	7,168	2,106 (29.4)	20.772	0.000	2,168 (30.3)	55.484	0.000	1,224 (17.1)	0.022	0.883	1,600 (22.3)	7.413	0.006
High school	7,176	1,864 (26.0)			1,772 (24.7)			1,232 (17.2)			1,468 (20.5)		
Region													
City	10,964	3,006 (27.4)	1.572	0.210	3,002 (27.4)	0.178	0.673	1,804 (16.5)	14.644	0.000	2,280 (20.8)	9.744	0.002
Rural	3,380	964 (28.5)			938 (27.8)			652 (19.3)			788 (23.3)		
Father Vocations													
Civil servant	4,662	1,240 (26.6)	5.133	0.077	1,220 (26.2)	8.112	0.017	964 (20.7)	2.635	0.268	930 (20.0)	8.746	0.013
Staff	6,762	1,928 (28.5)			1,930 (28.5)			1,180 (17.5)			1,502 (22.2)		
Other jobs	2,920	802 (27.5)			790 (27.1)			512 (17.5)			636 (21.8)		
Mother Vocations													
Civil servant	3,690	1,034 (28.0)	2.525	0.283	1,006 (27.3)	3.030	0.220	602 (16.3)	4.205	0.122	776 (21.0)	1.606	0.448
Staff	6,826	1,848 (27.1)			1,842 (27.0)			1,162 (17.0)			1,446 (21.2)		
Other jobs	3,828	1,088 (28.4)			1,092 (28.5)			692 (18.1)			846 (22.1)		
Father educational background													
Junior high school and below	6,980	1,944 (27.9)	6.013	0.049	1,976 (28.3)	9.487	0.009	1,268 (18.2)	12.469	0.002	1,566 (22.4)	11.276	0.004
High school	4,834	1,286 (26.6)			1,250 (25.9)			758 (15.7)			960 (19.9)		
University and above	2,530	740 (29.3)			714 (28.2)			430 (17.0)			542 (21.4)		
Mother educational background													
Junior high school and below	7,610	2,128 (28.0)	0.767	0.682	2,160 (28.4)	7.748	0.021	1,356 (17.8)	10.955	0.004	1,686 (22.2)	6.932	0.031
High school	4,444	1,210 (27.2)			1,158 (26.1)			760 (17.1)			930 (20.9)		
University and above	2,290	632 (27.6)			622 (27.2)			340 (14.9)			452 (19.7)		
Monthly family income													
<2,000	2,342	666 (28.4)	3.998	0.262	676 (28.9)	4.082	0.253	468 (20.0)	20.074	0.000	554 (23.7)	9.062	0.028
2,001–5,000	5,622	1,504 (26.8)			1,502 (26.7)			972 (17.3)			1,192 (21.2)		
2,001–8,000	3,846	1,086 (28.2)			1,056 (27.5)			614 (16.0)			802 (20.9)		
>8,000	2,534	714 (28.2)			706 (27.9)			402 (15.9)			520 (20.5)		
Grip strength													
≤P25	3,690	1,044 (28.3)	1.939	0.585	1,038 (28.1)	5.977	0.113	622 (16.9)	3.449	0.327	802 (21.7)	0.357	0.949
P26–50	3,502	964 (27.5)			998 (28.5)			574 (16.4)			744 (21.3)		
P51–75	3,584	1,002 (28.0)			938 (26.2)			618 (17.2)			762 (21.3)		
>P75	3,568	960 (27.0)			966 (27.1)			642 (18.0)			760 (21.3)		
Sit-ups													
≤P25	4,116	1,188 (28.9)	4.185	0.242	1,150 (27.9)	1.290	0.731	726 (17.6)	1.558	0.669	918 (22.3)	2.939	0.401
P26–50	3,300	890 (27.0)			892 (27.0)			550 (16.7)			692 (21.0)		
P51–75	3,734	1,020 (27.3)			1,036 (27.8)			644 (17.3)			790 (21.2)		
>P75	3,194	872 (27.3)			862 (27.0)			536 (16.8)			668 (20.9)		
Standing long jump													
≤P25	3,614	1,060 (29.3)	13.308	0.004	1,020 (28.2)	4.509	0.211	626 (17.3)	6.045	0.109	796 (22.0)	1.837	0.607
P26–50	3,634	1,040 (28.6)			1,018 (28.0)			592 (16.3)			770 (21.2)		
P51–75	3,544	920 (26.0)			928 (26.2)			588 (16.6)			736 (20.8)		
>P75	3,552	950 (26.8)			974 (27.4)			650 (18.3)			766 (21.6)		
Muscle Strength Index													
≤P25	3,582	1,052 (29.4)	9.491	0.023	996 (27.8)	5.032	0.169	640 (17.9)	24.372	0.000	824 (23.0)	23.417	0.000
P26–50	3,590	996 (27.7)			1,020 (28.4)			660 (18.4)			826 (23.0)		
P51–75	3,590	986 (27.5)			988 (27.5)			638 (17.8)			728 (20.3)		
>P75	3,582	936 (26.1)			936 (26.1)			518 (14.5)			690 (19.3)		

Table 2 shows the logistic regression analysis of psychological symptoms of Chinese adolescents. The results show that Chinese adolescents with a standing long jump score <P25 have a higher risk of psychological symptoms than those with a standing long jump score >P75 (OR = 1.14, 95% CI [1.03–1.26], *p* = 0.02). A similar result was found for the overall muscle strength index (OR = 1.18, 95% CI [1.06–1.30], *p* < 0.001).

**Table 2:** The logistic regression analysis of psychological symptoms for Chinese adolescents.

Independent variable	*β* value	Standard error	*Wald χ*2	*p*-value	OR (95% CI)
Gender					
Boys	0.01	0.04	0.11	0.743	1.01 [0.94–1.09]
Girls					1.00
Grade					
Junior high school	0.17	0.04	20.75	0.000	1.19 [1.10–1.28]
High school					1.00
Region					
Rural	0.06	0.04	1.57	0.21	1.06 [0.97–1.15]
City					1.00
Father Vocations					
Other jobs	0.04	0.05	0.69	0.41	1.05 [0.94–1.16]
Staff	0.10	0.04	5.04	0.03	1.10 [1.01–1.20]
Civil servant					1.00
Mother Vocations					
Other jobs	0.02	0.05	0.15	0.70	1.02 [0.92–1.13]
Staff	−0.05	0.05	1.08	0.30	0.95 [0.87–1.04]
Civil servant					1.00
Father educational background					
Junior high school and below	−0.07	0.05	1.79	0.18	0.93 [0.85–1.03]
High school	−0.13	0.05	5.83	0.02	0.88 [0.79–0.98]
University and above					1.00
Mother educational background					
Junior high school and below	0.02	0.05	0.12	0.73	1.02 [0.92–1.13]
High school	−0.02	0.06	0.10	0.75	0.98 [0.88–1.10]
University and above					1.00
Monthly family income					
<2,000	0.01	0.06	0.04	0.84	1.01 [0.89–1.15]
2,001–5,000	−0.07	0.05	1.79	0.18	0.93 [0.84–1.03]
2,001–8,000	0.00	0.06	0.00	0.96	1.00 [0.90–1.12]
>8,000					1.00
Grip strength					
≤P25	0.07	0.05	1.75	0.19	1.07 [0.97–1.19]
P26–50	0.03	0.05	0.34	0.56	1.03 [0.93–1.15]
P51–75	0.05	0.05	0.99	0.32	1.05 [0.95–1.17]
>P75					1.00
Sit-ups					
≤P25	0.08	0.05	2.17	0.14	1.08 [0.98–1.20]
P26–50	−0.02	0.06	0.09	0.76	0.98 [0.88–1.10]
P51–75	0.00	0.05	0.00	0.99	1.00 [0.90–1.11]
>P75					1.00
Standing long jump					
≤P25	0.13	0.05	5.93	0.02	1.14 [1.03–1.26]
P26–50	0.09	0.05	3.15	0.08	1.10 [0.99–1.22]
P51–75	−0.04	0.05	0.57	0.45	0.96 [0.86–1.07]
>P75					1.00
Muscle Strength Index					
≤P25	0.16	0.05	9.36	0.00	1.18 [1.06–1.30]
P26–50	0.08	0.05	2.37	0.12	1.09 [0.98–1.21]
P51–75	0.07	0.05	1.63	0.20	1.07 [0.96–1.19]
>P75					1.00

**Figure 2:**
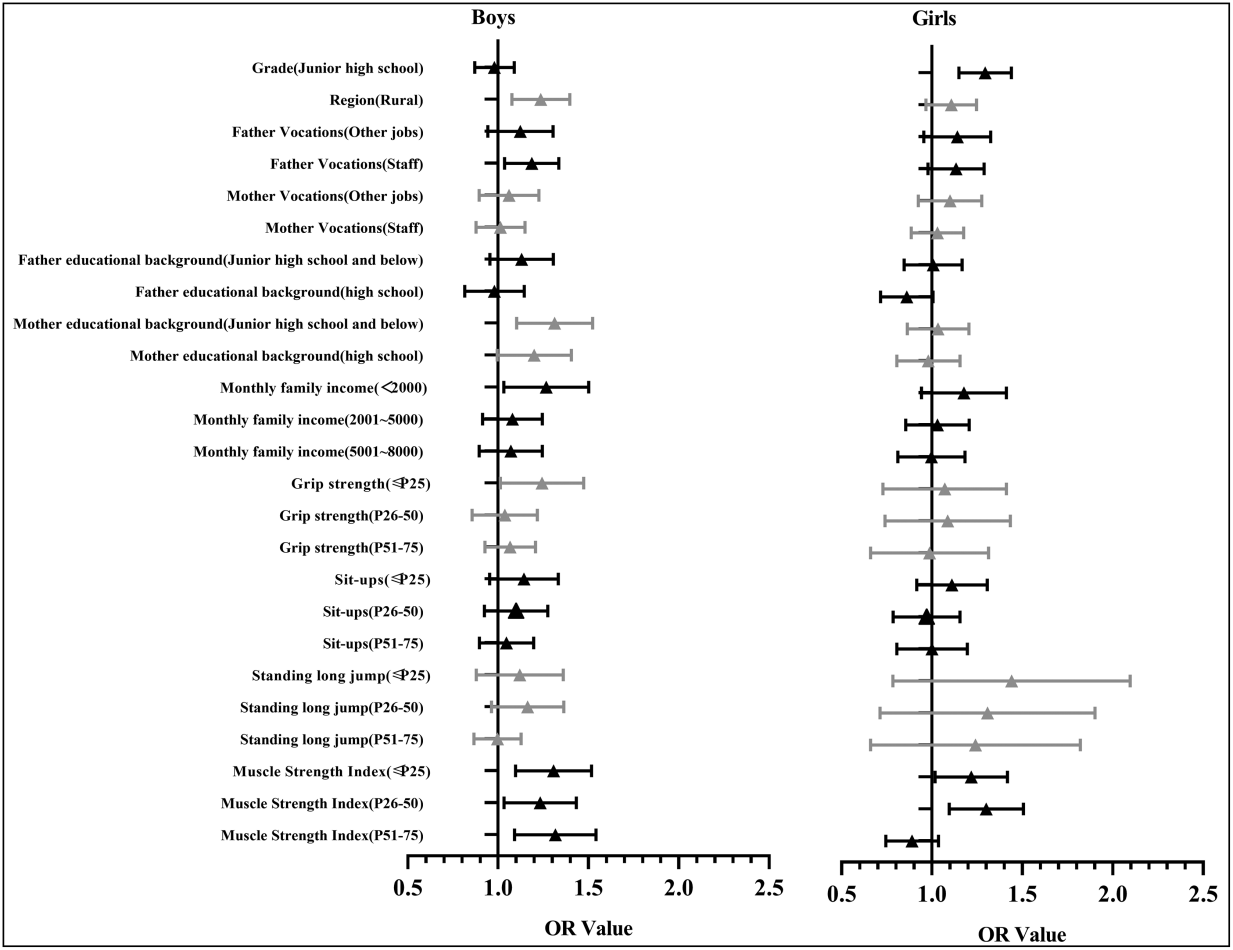
The diagram of the OR value (95% CI) of regression analysis of the psychological symptoms and the muscle strength of Chinese adolescents.

A logistic regression analysis of psychological symptoms was also conducted after stratification by gender (Table 3). The results show that males with a grip strength ≤P25 (OR = 1.23, 95% CI [1.02–1.48], *p* = 0.03) and a muscle strength index ≤P25 (OR = 1.30, 95% CI [1.10–1.52], *p* < 0.001), P26–50 (OR = 1.22, 95% CI [1.04–1.44], *p* = 0.02) or P51–75 (OR = 1.30, 95% CI [1.10–1.55], *p* < 0.001) have a higher risk of psychological symptoms than those with a muscle strength index >P75. Females with a muscle index ≤P25 (OR = 1.21, 95% CI [1.02–1.42], *p* = 0.03) or P26–50 (OR = 1.29, 95% CI [1.10–1.51], *p* < 0.001) have a higher risk of psychological symptoms than those with a muscle strength index >P75. Figure 2 shows a diagram of the OR value (95% CI) for the regression analysis of the psychological symptoms of Chinese adolescents.

**Table 3:** The logistic regression analysis of psychological symptoms for Chinese adolescents after stratification by gender.

Independent variable	Boys		Girls		Boys compared to girls	
	*p*	OR (95% CI)	*p*	OR (95% CI)	*p*	OR (95% CI)
Grade						
Junior high school	0.69	0.98 [0.87–1.09]	0.00	1.29 [1.15–1.44]	0.41	0.96 [0.85–1.07]
High school		1.00		1.00	0.00	1.26 [1.12–1.41]
Region						
Rural	0.00	1.23 [1.08–1.40]	0.15	1.10 [0.97–1.25]	0.03	1.19 [1.01–1.39]
City		1.00		1.00	0.18	1.07 [0.97–1.17]
Father Vocations						
Other jobs	0.18	1.11 [0.95–1.31]	0.14	1.13 [0.96–1.33]	0.51	1.06 [0.89–1.27]
Staff	0.01	1.18 [1.04–1.34]	0.08	1.13 [0.98–1.29]	0.04	1.13 [1.01–1.26]
Civil servant		1.00		1.00	0.31	1.08 [0.93–1.24]
Mother Vocations						
Other jobs	0.50	1.05 [0.90–1.23]	0.28	1.09 [0.93–1.28]	0.36	1.07 [0.92–1.25]
Staff	0.93	1.01 [0.88–1.15]	0.76	1.02 [0.89–1.18]	0.13	1.10 [0.98–1.23]
Civil servant		1.00		1.00	0.19	1.11 [0.95–1.31]
Father educational background						
Junior high school and below	0.14	1.12 [0.96–1.31]	1.00	1.00 [0.85–1.17]	0.08	1.11 [0.99–1.24]
High school	0.73	0.97 [0.82–1.15]	0.06	0.85 [0.72–1.01]	0.10	1.12 [0.98–1.30]
University and above		1.00		1.00	0.87	0.99 [0.81–1.19]
Mother educational background						
Junior high school and below	0.00	1.30 [1.11–1.53]	0.79	1.02 [0.87–1.21]	0.01	1.15 [1.03–1.28]
High school	0.06	1.19 [1.00–1.41]	0.73	0.97 [0.81–1.16]	0.17	1.11 [0.96–1.28]
University and above		1.00		1.00	0.34	0.90 [0.74–1.11]
Monthly family income						
<2,000	0.02	1.25 [1.04–1.51]	0.14	1.16 [0.95–1.42]	0.23	1.12 [0.93–1.36]
2,001–5,000	0.39	1.07 [0.92–1.25]	0.80	1.02 [0.86–1.21]	0.18	1.09 [0.96–1.24]
2,001–8,000	0.51	1.06 [0.90–1.25]	0.87	0.98 [0.82–1.19]	0.15	1.12 [0.96–1.31]
>8,000		1.00		1.00	0.68	1.04 [0.86–1.27]
Grip strength						
≤P25	0.03	1.23 [1.02–1.48]	0.84	1.03 [0.75–1.43]	0.01	1.27 [1.05–1.53]
P26–50	0.79	1.03 [0.86–1.22]	0.79	1.05 [0.76–1.45]	0.64	1.05 [0.87–1.25]
P51–75	0.38	1.06 [0.93–1.21]	0.78	0.95 [0.68–1.33]	0.04	1.19 [1.01–1.40]
>P75		1.00		1.00	0.70	1.07 [0.77–1.47]
Sit-ups						
≤P25	0.14	1.13 [0.96–1.34]	0.30	1.10 [0.92–1.31]	0.26	1.09 [0.94–1.28]
P26–50	0.30	1.09 [0.93–1.28]	0.67	0.96 [0.79–1.16]	0.03	1.20 [1.02–1.42]
P51–75	0.58	1.04 [0.9–1.20]	0.88	0.99 [0.81–1.20]	0.17	1.12 [0.95–1.32]
>P75		1.00		1.00	0.54	1.06 [0.88–1.27]
Standing long jump						
≤P25	0.38	1.1 [0.89–1.37]	0.22	1.34 [0.84–2.14]	0.41	1.10 [0.88–1.37]
P26–50	0.11	1.15 [0.97–1.37]	0.41	1.22 [0.76–1.94]	0.01	1.27 [1.06–1.51]
P51–75	0.88	0.99 [0.87–1.13]	0.57	1.15 [0.71–1.86]	0.11	1.15 [0.97–1.37]
>P75		1.00		1.00	0.22	1.34 [0.84–2.13]
Muscle Strength Index						
≤P25	0.00	1.30 [1.10–1.52]	0.03	1.21 [1.02–1.42]	0.60	1.04 [0.89–1.22]
P26–50	0.02	1.22 [1.04–1.44]	0.00	1.29 [1.10–1.51]	0.28	0.92 [0.79–1.07]
P51–75	0.00	1.30 [1.10–1.55]	0.13	0.88 [0.75–1.04]	0.00	1.43 [1.22–1.69]
>P75		1.00		1.00	0.72	0.97 [0.82–1.15]

**Figure 3:**
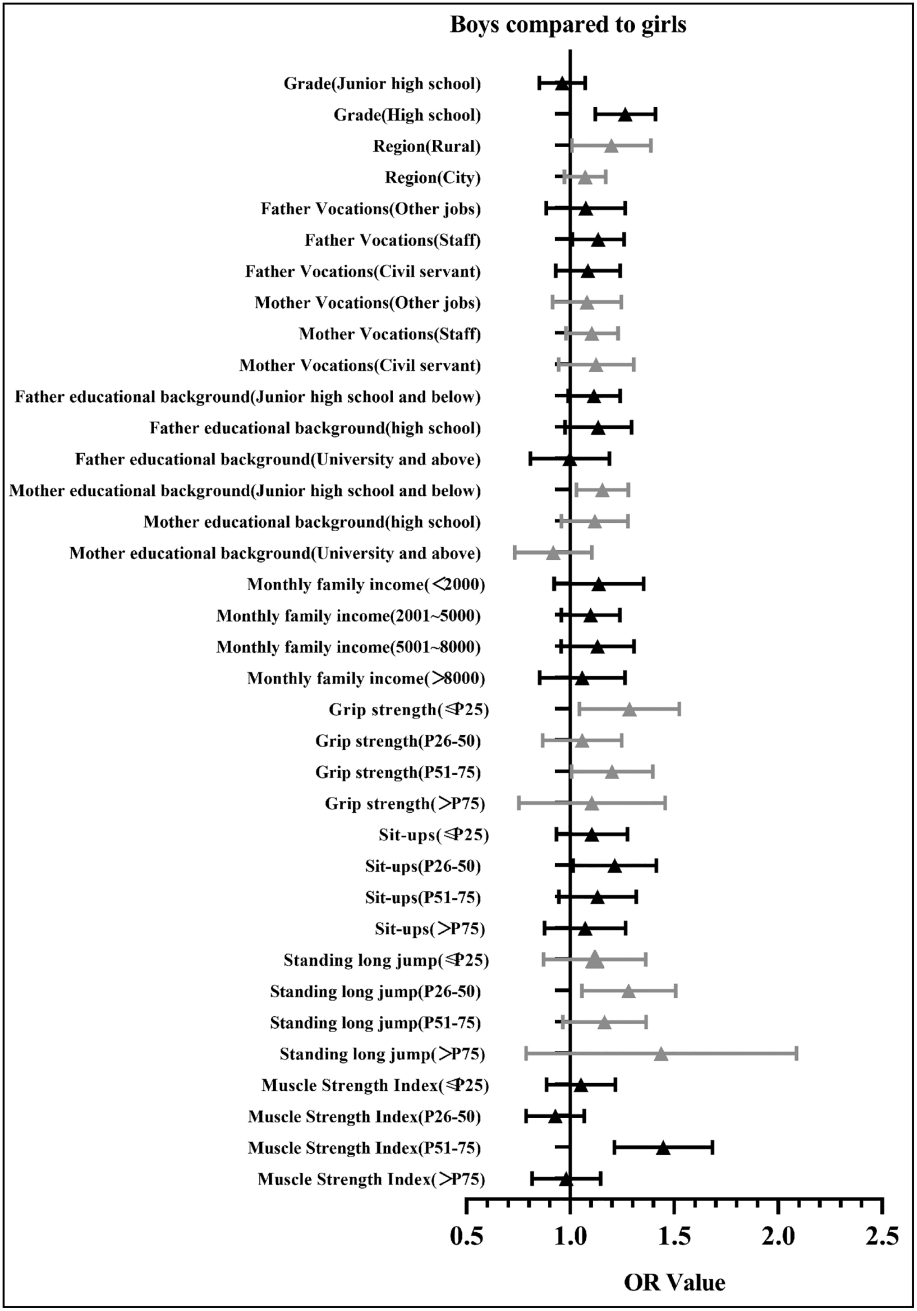
The diagram of the OR value (95% CI) of regression analysis of the psychological symptoms and the muscle strength of Chinese adolescents (in comparison with girls).

Figure 3 shows the diagram of the OR value (95% CI) for the regression analysis of psychological symptoms for Chinese males. Compared with females, the OR values for males in most groups were over one (OR = 1.04–1.43), indicating that males have a higher risk of psychological symptoms than females and that psychological symptoms are more correlated to muscle strength for males than females.

## Discussion

The present study used a large sample of Chinese adolescents to explore the correlation between muscle strength and psychological symptoms, concluding that the detection rate of psychological symptoms for Chinese adolescents with a muscle strength index >P75 (19.26%) was significantly lower than among those with a muscle strength index ≤P25 (23.00%) (χ^2^ = 23.417, *P* < 0.01). Compared with females, the OR values for males in most groups were over one (OR = 1.04–1.43), indicating that males have a higher risk of psychological symptoms than females.

According to this study, 21.39% of Chinese teenagers had psychological symptoms. These results are higher than those of Tao et al. (2020) (11.2%) and lower than those of Liang et al. (2016) (22.9%) (Tao et al., 2020; Liang, 2016). These differences in results are likely due to differences in survey times and the recall ability of the students surveyed. Otherwise, the high prevalence of psychological symptoms among Chinese adolescents needs to be concerned.

When divided by demographics, male junior high school students had a higher detection rate of psychological symptoms than female students, which is in concordance with the result of Wu (2019). It was reported that male teenagers typically have more bad health habits than female teenagers, such as more time spent playing online games or watching videos (Zapata-Lamana et al., 2021), smoking (Skvortcova & Lushkina, 2018), and drinking (Chung et al., 2018), resulting in a higher rate of psychological symptoms (Silva et al., 2021). The detection rate of psychological symptoms of junior high school students was higher than that of senior high students, which can be attributed to the fact that junior high school students are in a rather unstable state of mind at the peak of their puberty, while high school students have better psychological knowledge and can better adjust to their psychological state (Zhang et al., 2020a). Our study also found a higher rate of psychological symptoms among students in the country regions compared with urban students, which can be explained by disparities in educational resources and emphasis on psychological health between rural and urban schools (Chen et al., 2019). Rural students are also more likely to have parents with lower educational levels than urban students and there is likely less emphasis placed on mental health in these homes. This implies that the higher the academic level of the parents, the lower the incidence of psychological symptoms among the students, which is also the conclusion of Kandola et al. (2020). The hypothesis is that parents with higher levels of education are more aware of the importance of psychological health and how to get help when their children have psychological symptoms (Ekelund et al., 2016). Another finding of our study is that the higher the family income, the lower the incidence of psychological symptoms among Chinese teenagers. Family income is correlated to the educational attainment of the parents, corresponding with a better emphasis on mental health and more access to resources when help is needed (Tang et al., 2021). These results indicate that more emphasis should be placed on the psychological development and mental health of rural students and on cultivating a better environment for the psychological development of these students.

Our study found that Chinese adolescents with a lower percentile of muscle index (*e.g.*, <P25, or P26–50) have a higher risk of psychological symptoms than those with a higher muscle strength index (*e.g.*, >P75). In one study (Zhang et al., 2020b), the students whose muscle strength levels were higher had better physical activity levels and performed better in teamwork and socializing, both of which play a positive role in lowering psychological symptoms. Another study (Szeremeta, Grzywacz & Czarny, 2020) found that students with higher muscle strength levels spent more time participating in extracurricular sports and they usually communicated better with their partners, two things that also have a positive impact on lowering psychological symptoms.

We also found that compared with females, the muscle strength of males is a more significant factor in the presence of psychological symptoms. Males always perceive several benefits to becoming more muscular and they wanted to be more muscular because they believe that it would make them feel more confident and more attractive to women (Frederick et al., 2007). However, masculinities are often associated with poor mental health and well-being outcomes for men (Clark et al., 2020) and they are reluctant to seek professional psychological help because of the traditional ideas of masculinity that being stronger and self-reliant (Staiger et al., 2020). This vicious cycle implies that more attention should be put on the muscle strength level and psychological symptoms of males.

## Strengths and limitations

Using a large national sample, this study is the first to analyze the correlation between the muscle strength of Chinese adolescents and the presence of psychological symptoms. This study also has some limitations. This is a cross-sectional survey, therefore, it was capable of identifying correlation but not causation. Longitudinal studies are needed to explore the causal relationship between psychological symptoms and muscle strength in Chinese adolescents. We also used self-evaluated questionnaires to estimate psychological symptoms, which are inherently limited by the memory recall ability of the participating students.

## Conclusions

Our original study found that the presence of psychological symptoms in Chinese adolescents is correlated with muscle strength. For males, the correlation between psychological symptoms and muscle strength was stronger than for females. The significance of the present study is that it provides insights into the importance of integrated mental and physical fitness intervention strategies that promote muscle strength and psychological symptoms in Chinese adolescents simultaneously. Targeted intervention and guidance for males and females should be separated for better psychological development.

## Supplemental Information

10.7717/peerj.14133/supp-1 Supplemental Information 1 Raw data.

## Additional Information and Declarations

### Data Availability

The following information was supplied regarding data availability: The raw measurements are available in the Supplemental File.
